# Featurization strategies of supplementary cementitious materials for enhancing interpretability and precision in machine learning based concrete strength prediction

**DOI:** 10.1038/s41598-026-57522-1

**Published:** 2026-06-11

**Authors:** Jeonghyun Kim, Diana Bajare, Donwoo Lee

**Affiliations:** 1 Institute of Sustainable Building Material and Engineering Systems, Faculty of Civil and Mechanical Engineering, Riga Technical University, Kipsalas Iela 6a, Riga, 1048 Latvia; 2 School of Industrial Design & Architectural Engineering, Korea University of Technology & Education, 1600 Chungjeol-ro, Byeongcheon-Myeon, Cheonan, 31253 Republic of Korea

**Keywords:** Supplementary cementitious materials, Machine learning, Variable strategy, Curse of dimensionality, SHAP analysis, Strength prediction, Engineering, Materials science, Mathematics and computing

## Abstract

This study investigates the impact of input variable processing methods for supplementary cementitious materials (SCMs) on the performance of machine learning (ML)-based concrete strength prediction models. SCMs possess unique pozzolanic and latent hydraulic mechanisms. However, it has not been clearly established whether it is more advantageous to treat variables individually to capture SCM characteristics or to aggregate them into a single variable to avoid the curse of dimensionality. This study compared and analyzed the predictive performance between strategies of individualizing versus combining SCM components using 13 databases. Furthermore, SHAP analysis was used to interpret the physical impact of each strategy on the internal decision-making mechanisms of ML models. The results showed that, in most cases, the individualized model recorded superior accuracy compared to the combined model. Particularly in environments where the strength contribution of each SCM component varied in direction, variable separation prevented the information cancel-out effect, allowing the model to capture physical causality more precisely. Conversely, in simple environments, where the influence of a specific variable such as age was overwhelming or the entire SCM exhibited a uniform trend in strength development, combining SCMs as a single variable could be advantageous for stable performance.

**Supplementary Information:**

The online version contains supplementary material available at 10.1038/s41598-026-57522-1.

## Introduction

As the AI era unfolds with the integration of artificial intelligence across various industrial sectors, the application of machine learning (ML) technologies in concrete engineering is rapidly increasing. As an alternative to traditional experimental approaches, the ML-based prediction of mechanical properties has become an effective tool for significantly reducing the immense human and temporal resources required during the mix design process. However, several issues remain regarding the practical applicability and reliability of these ML models. In particular, with the recent increase in demand for eco-friendly concrete, there is a rapid surge in utilizing not only traditional supplementary cementitious materials (SCMs), such as ground granulated blast furnace slag (GGBFS), fly ash (F-ash), and silica fume (SF), but also industrial by-products including waste granite powder, municipal solid waste ash, sewage sludge ash, and construction waste powder^1–3^. This diversification of SCM types adds complexity to modeling due to the unique physicochemical properties and pozzolanic reaction mechanisms inherent in each SCM^4,5^. In the ML modeling process, treating these diverse SCM types as separate input variables increases the model dimensionality, which may lead to reduced computational efficiency and overfitting, a phenomenon known as the curse of dimensionality. To circumvent these issues and simplify model structure, some existing studies have adopted an approach of aggregating different SCMs into a single, combined variable. For instance, a recent study^6^ developed an ML model to predict CO_2_ emissions based on concrete mix designs where SCMs were integrated. The dataset for that model included SCMs with vastly different properties, such as natural volcanic pozzolan^7^, F-ash and limestone powder (LP)^8^, sewage sludge ash^9^, SF, and marble powder (MP)^10^. Conversely, attempts have been made to use the oxide composition of SCMs as input variables to precisely reflect individual SCM characteristics for predicting concrete strength. While this allows for a comprehensive understanding of how various chemical components affect predictive performance, it has been pointed out that such granular approaches may be limited by training datasets that do not encompass the full diversity of existing SCMs. This, in turn, may restrict the versatility of the constructed models and the generalizability of research findings^11^.

Despite these ongoing efforts to develop robust models, there remains a significant lack of systematic comparative research on whether separating SCMs into independent variables or aggregating them into a total SCM content is more advantageous for predicting concrete performance. In this context, the research problem addressed in this study is how to effectively manage diverse SCM types with differing physicochemical properties within ML models. Given that modern concrete technology frequently involves new types of SCMs and complex ternary binder systems, this investigation is essential. Therefore, rather than an attempt to develop a new machine learning algorithm, this work can be fundamentally viewed as an applied feature engineering study aimed at establishing a variable utilization strategy. Accordingly, this study quantitatively compares the effects of SCM variable processing methods on the accuracy and interpretability of concrete strength prediction models using 13 databases.

To achieve these objectives, this study first briefly summarizes the characteristics of representative SCMs and their effects on concrete strength through a literature review. Subsequently, databases obtained from literature were collected and refined to ensure suitability for ML modeling. Based on the processed data, a comparative analysis was performed to evaluate the predictive accuracy between the approach of using individual SCMs as separate input variables and the strategy of treating them as a single integrated variable. Furthermore, post-hoc analysis using SHapley Additive exPlanations (SHAP) was conducted for each modeling scenario to elucidate how different SCM processing methods influence the models decision-making mechanisms.

## Characteristics of SCMs and their effects on concrete performance

In this section, the SCMs widely used as cement replacements, such as GGBFS, F-ash, SF, and Metakaolin (MK), were selected for brief review, as they constitute the components of the databases collected for this study.

GGBFS exhibits latent hydraulicity due to its relatively high CaO content and leads to long-term strength development through activation by Ca(OH)_2_, a hydration product of cement. In contrast, SF, F-ash, and MK contribute to strength development primarily through pozzolanic reactions. In particular, SF and nano/micro-silica (NS/MS) maximize early-stage pozzolanic reactions due to their high-purity amorphous silica and fine particle sizes^12,13^. Conversely, F-ash consumes free lime at a relatively slower rate, contributing to the long-term densification of the hardened matrix structure^14^. Table 1 summarizes the physicochemical compositions of each SCM reported in the literature, offering detailed insights into their material properties.

**Table 1:** Physical and chemical characteristics of selected SCMs^15^**.**

Type	Chemical composition (%)				Specific area (m^2^/kg)
	SiO_2_	Al_2_O_3_	Fe_2_O_3_	CaO	
GGBFS	32.3–46.1	3.4–16.3	0.4–1.0	35.3–43.2	384–800
SF	87.3–96.2	0.3–0.6	0.2–0.6	0.1–3.2	14,200–27,300
F-ash	30.6–46.4	13.9–18.8	5.3–8.3	21.6–28.9	285–375
MK	50.8–65.5	32.4–41.1	0.4–4.3	0–2.4	4370–20,000
NS/MS	> 98	0–0.3	0–0.3	0.1–0.6	30,000–954,000

Due to the distinct physicochemical properties of each SCM, their influence on concrete strength exhibits highly non-linear patterns depending on the SCM type and replacement ratio. For example, SF positively influences the mechanical properties of concrete by filling micropores and enhancing the microstructure. Previous studies indicate that compressive strength (CS) increases with SF replacement up to 15–25%, beyond which it tends to decrease^16^. In the case of GGBFS, although reported optimal dosages vary by source, it generally exhibits peak performance at replacement ratios between 20 55%; beyond this range, the strength enhancement diminishes^17,18^. When replacing cement at low levels (up to 30%), F-ash can achieve higher strength than conventional concrete through long-term pozzolanic reactions without a significant loss in early-age strength^19^. The optimal replacement ratio is typically reported within the 10–30% range, exceeding 30–50% markedly slows the hydration rate, negatively affecting strength development^20^. MK, a highly reactive pozzolan obtained by calcining kaolinite clay, contributes to strength enhancement at a replacement ratio of approximately 15–20%, after which the CS tends to decline^21^. Regarding limestone powder (LS), some studies have reported that incorporating LS at a level of approximately 15% can result in CS superior to that of conventional cement-based concrete^22^.

SCMs are promising materials for enhancing concrete properties and reducing CO_2_ emissions from the cement industry. However, due to these differing chemical components and hydration rates, grouping these SCMs into a single total SCM variable can trigger an information cancel-out effect. When SCMs with completely opposite effects, such as SF which increases early strength and F-ash which acts slowly, are combined into one input, the ML model may struggle to distinguish their individual roles. Consequently, the unique chemical signals of each SCM collapse into a simple average trend, and their opposing physical influences neutralize each other. These non-linear correlations underscore the risk that ML models may fail to capture essential material behaviors if trained solely on total SCM content, thereby necessitating the investigation into SCM featurization strategies in this study.

## Machine learning

### Database collection and composition

In this study, a total of 13 databases were collected from the literature. A prerequisite for selecting these databases was the inclusion of at least two types of SCMs as independent input variables. This criterion was established to objectively compare the effects of different SCM variable processing strategies. After selecting the databases, a data cleaning process was performed to prevent model overfitting and enhance data quality. Within each of the 13 databases, duplicate mixture entries that were redundantly recorded were removed. Duplicate entries were defined as exact cross-column matches where all input mixture proportions and the final output values were identical across rows. Near-duplicate entries with minor variance in target values were deliberately preserved to capture multi-source laboratory variations. For DB4, the number of records was significantly reduced from 3299 raw points to 330. This substantial reduction is attributed to the characteristics of the original DB4, which was constructed to predict the shrinkage behavior of concrete mixtures. Consequently, for a single concrete mixture sharing identical mix proportions and strength, approximately 6 to 16 shrinkage data points were recorded. Since the objective of this study is to examine the impact of SCM variables in prediction models, utilizing these datasets without refinement would cause the model to repeatedly learn identical input–output pairs, leading to unreliable predictions. Thus, a filtering process was conducted during preprocessing to remove these redundant entries, resulting in a nearly 90% reduction in the total records for DB4. Subsequently, a feature selection process was conducted to exclude noise variables that could impair learning efficiency. For instance, while DB4 included curing temperature, the values were predominantly distributed within a narrow range of 20 to 23 °C. Given the margin of error in experimental equipment and procedures, such variables may act as constant. These near-zero variance features were excluded from the final set of input variables, as they do not provide meaningful information. It is worth noting that this study did not involve the removal of statistical outliers. Instead, the natural variability inherent in multi-source experimental data was deliberately preserved. By allowing the model to learn from these authentic fluctuations rather than a sanitized dataset, the study aimed to realistically evaluate the effect of SCM featurization methods under actual conditions of data uncertainty. Detailed information regarding the sources, sample sizes, and input variables for each database is summarized in Table 2, and the full dataset is provided in the Appendix A.

**Table 2:** Information on selected database.

No	Source	Raw data point	Processed data point	Types of SCM*	Input**	Output***
DB1	^23^	188	185	F-ash, GGBFS, MK	C, B, W, w/b, SP, VMA, NCA20, NCA10, RCA20, RCA10, Sand, Age	CS
DB2	^24^	1030	1005	F-ash, GGBFS	C, W, SP, CA, FA, Age	CS
DB3	^25^	810	792	S, SF, LP, QP, F-ash, NS	C, A, W, Fi, SP, T, Age	CS
DB4	^26^	3299	330	F-ash, S,MK, BA, G, LP, Filler, SS, GP, NP, MS	w/c, a/c, FA, CA, C, S-fi, SP	CS
DB5	^27^	229	229	F-ash, SF, MP	C, FA, CA, W, SP, Age	CS
DB6	^27^	229	229	F-ash, SF, MP	C, FA, CA, W, SP, Age	TS
DB7	^28^	980	976	F-ash, GGBFS, SF, CC	C, NCA, RCA, NFA, RFA, W, SP, w/b	CS
DB8	^28^	980	307	F-ash, GGBFS, SF	C, NCA, RCA, NFA, RFA, W, SP, w/b	EM
DB9	^28^	980	457	F-ash, GGBFS, SF, CC	C, NCA, RCA, NFA, RFA, W, SP, w/b	FS
DB10	^29^	1456	1451	F-ash, SF, GGBFS	C, W, CA, FA, SP	CS
DB11	^29^	1456	280	F-ash, SF, GGBFS	C, W, CA, FA, SP	FS
DB12	^29^	1456	203	F-ash, SF, GGBFS	C, W, CA, FA, SP	EM
DB13	^29^	1456	394	F-ash, SF, GGBFS	C, W, CA, FA, SP	TS

*QP, quartz powder; BA, bottom ash; G, gymsum; F, filler, SS, steel slag; GP, glass powder; NP, natural pozzolan; MP, marble powder; CC, calcined clay.

**C, cement; B, binder; W, water; w/b, water-to-binder ratio; NCA20, natural coarse aggregate (10–20 mm); NCA10, natural coarse aggregate (0–10 mm); RCA20, recycled coarse aggregate (10–20 mm); RCA10, recycled coarse aggregate (0–10 mm); CA, coarse aggregate; FA, fine aggregate; A, aggregate; Fi, fiber; T, temperature.

***TS, tensile strength; EM, elastic modulus; FS; flexural strength.

### SCM featurization strategies

Two distinct ML scenarios were applied to each database. The first scenario is the ‘individual SCMs model,’ in which each SCM component is assigned as an independent input variable. This approach offers the advantage of allowing the model to separately learn the unique physicochemical characteristics of each material. However, it expands the dimensionality of the input variables as the variety of SCMs increases. The second is the ‘combined SCM model,’ which reduces the dimensionality by summing the all individual SCMs into a single variable (Total SCM content). This strategy is expected to lower model complexity by minimizing the number of features.

### Machine learning modeling and interpretation

To effectively capture the complex and non-linear behavior of concrete, this study adopted the Extreme Gradient Boosting (XGBoost), a tree-based ensemble algorithm widely recognized for its robust predictive performance for concrete properties. To ensure that the observed findings were not specific to a single boosting architecture, the Random Forest (RF) algorithm, a bagging-based ensemble method, was also adopted. Contrasting these two fundamentally different ensemble structures allows for a robust validation of the SCM featurization strategy.

Each database was randomly partitioned into a training set (80%) and a test set (20%) for final model verification. To prevent weight bias arising from differing scales among variables, standardization was performed to transform all input features to have a mean of 0 and a standard deviation of 1. Within the training phase, Bayesian optimization was utilized to fine-tune key hyperparameters to efficiently identify the optimal parameter configuration while minimizing computational overhead^30,31^. The specific hyperparameter search ranges are detailed in Table 3. To isolate and precisely discuss the impact of the SCM featurization strategy, the identical optimization protocol and hyperparameter search bounds were applied equally to both the individualized and combined models across each database. Hyperparameters not explicitly specified in the table were kept at their default values provided by the software package.

**Table 3:** Hyperparameter search spaces for machine learning models.

Model	Hyperparameter	Search range
XGBoost	n_estimators	50, 400
	max_depth	1, 10
	learning_rate	0.01, 0
RF	n_estimators	50, 400
	max_depth	3, 15
	min_samples_split	2, 10

Furthermore, to eliminate potential bias stemming from a single random data partition and to further reinforce the validity of our findings, an additional validation scheme via repeated random train-test splitting was performed. Specifically, the validation process sequentially iterated random data shuffling based on five independent random seeds combined with fivefold cross-validation, ensuring that the models were evaluated over entirely fresh and independent test set compositions for each trial. The final models performance was quantitatively evaluated using four metrics: the coefficient of determination (R^2^), RMSE, mean absolute error (MAE), and mean absolute percentage error (MAPE). To verify the statistical significance of using SCMs as individualized variables compared to the combined approach, the Wilcoxon signed-rank test was performed.

After the ML modeling, the SHAP (Shapley Additive exPlanations) method was introduced to overcome the black-box nature of ML models and transparently interpret the impact of SCM featurization strategies. SHAP values quantitatively allocate the contribution of each input feature to the final prediction. As demonstrated in previous literature^32^, the SHAP method has properly captured the complex physicochemical influences of SCMs on concrete properties, providing reliable material-specific insights. Building upon this established capability, this study utilized SHAP to elucidate how individual SCM variables versus combined SCM variables influence strength development in terms of both directionality and importance.

All computational procedures were implemented using Python 3.13.9, utilizing the XGBoost (v3.2.0) and SHAP (v0.51.0) libraries.

## Results

The performance metrics of the ML models, based on the different SCM input variable processing strategies, are summarized in Table 4. Since the databases differ in their distributions and experimental conditions, the cross-database aggregation of results should be interpreted cautiously. The analysis in this study primarily focuses on the comparative impact of featurization within each dataset.

**Table 4:** Predictive performance of XGBoost models under two SCM scenarios.

No	Set	R^2^		RMSE		MAE		MAPE	
		Combined	Individual	Combined	Individual	Combined	Individual	Combined	Individual
DB1	Train	0.814	0.905	6.8	5.2	4.6	3.8	21.7	14.4
	Test	0.934	0.943	5.1	4.7	3.9	3.6	19.0	15.7
DB2	Train	0.904	0.904	4.9	4.9	3.4	3.3	11.6	11.1
	Test	0.918	0.93	4.9	4.5	3.3	3.0	11.5	10.6
DB3	Train	0.949	0.949	8.9	8.9	6.2	6.2	5.5	5.5
	Test	0.968	0.966	7.4	7.5	5.0	5.2	5.3	5.5
DB4	Train	0.795	0.844	11.9	10.5	8.2	7.6	15.6	14.2
	Test	0.804	0.862	12.6	10.6	8.8	7.3	16.2	12.9
DB5	Train	0.895	0.937	3.6	2.7	2.6	2.0	5.7	4.2
	Test	0.962	0.949	2.4	2.7	1.8	1.8	3.8	4.0
DB6	Train	0.875	0.928	0.31	0.24	0.23	0.19	4.93	4.05
	Test	0.901	0.922	0.33	0.29	0.23	0.21	4.75	4.29
DB7	Train	0.807	0.830	6.8	6.4	4.9	4.5	12.8	11.7
	Test	0.848	0.856	6.5	6.3	4.3	4.2	12.1	11.5
DB8	Train	0.766	0.763	3.9	3.9	2.9	3.0	9.6	9.8
	Test	0.834	0.847	3.4	3.2	2.3	2.2	8.0	7.6
DB9	Train	0.726	0.745	0.46	0.45	0.34	0.33	10.4	10.0
	Test	0.803	0.814	0.43	0.41	0.30	0.29	10.0	9.4
DB10	Train	0.755	0.835	8.7	7.1	6.0	5.0	15.0	12.4
	Test	0.804	0.854	8.2	7.0	5.9	4.9	15.5	12.5
DB11	Train	0.796	0.829	1.05	0.94	0.69	0.66	12.8	12.5
	Test	0.898	0.901	0.71	0.70	0.48	0.50	7.3	8.0
DB12	Train	0.658	0.677	4.9	4.7	3.3	3.2	12.1	11.6
	Test	0.551	0.599	5.7	5.4	4.3	4.0	12.5	11.5
DB13	Train	0.761	0.826	0.61	0.53	0.43	0.37	12.4	10.8
	Test	0.849	0.864	0.48	0.46	0.37	0.34	11.9	10.4

### Predictive performance

**Fig. 1:**
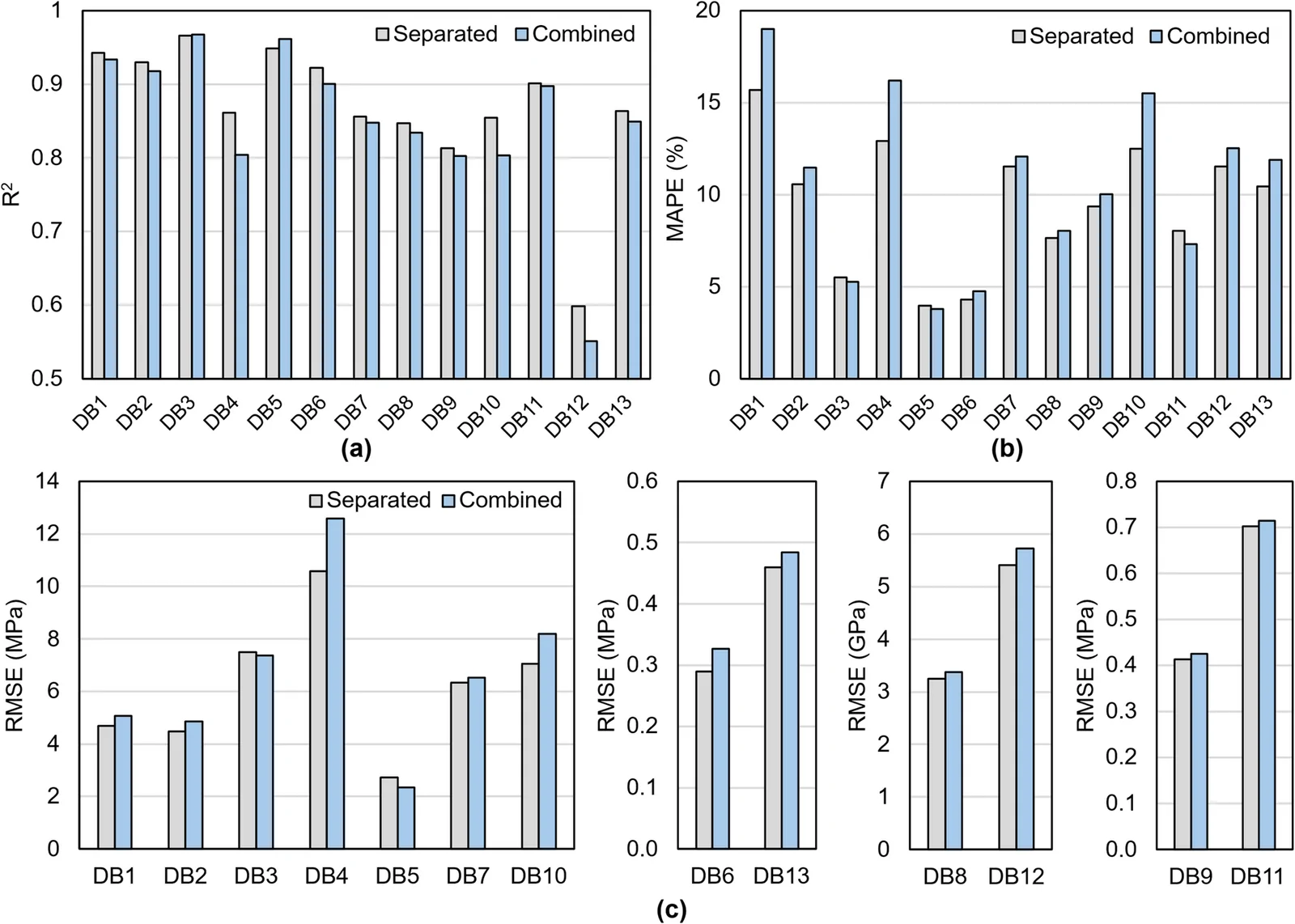
Comparison of predictive performance across databases using different SCM modeling approaches.

Figure 1 illustrates the R^2^, MAPE, and RMSE results of the test datasets for both the individual and combined SCM scenarios across the 13 databases. In nearly all cases, the individual SCM scenario exhibited superior predictive accuracy compared to the combined scenario.

Regarding the R^2^, most databases demonstrated higher explanatory power when SCMs were inputted individually. In particular, for DB10, the R^2^ improved from 0.804 in the combined approach to 0.854 in the separated approach. Similarly, a significant improvement in precision was observed for DB4, where the R^2^ increased from 0.804 to 0.862. This trend was consistently observed in both the training and test datasets, validating the robust generalization capability of the individual SCM models.

Error metrics, such as RMSE and MAPE, were also markedly lower in the individual SCM scenario. In the case of DB4, the RMSE decreased from 12.6 MPa in the combined scenario to 10.6 MPa with individual featurization, while the MAPE was reduced from 16.2 to 12.9%. Similarly, DB12, which focuses on the elastic modulus (EM), yielded more precise predictions under the individual SCM approach, with the RMSE and MAPE decreasing from 5.72 GPa to 5.41 GPa and from 12.5 to 11.5%, respectively.

**Fig. 2:**
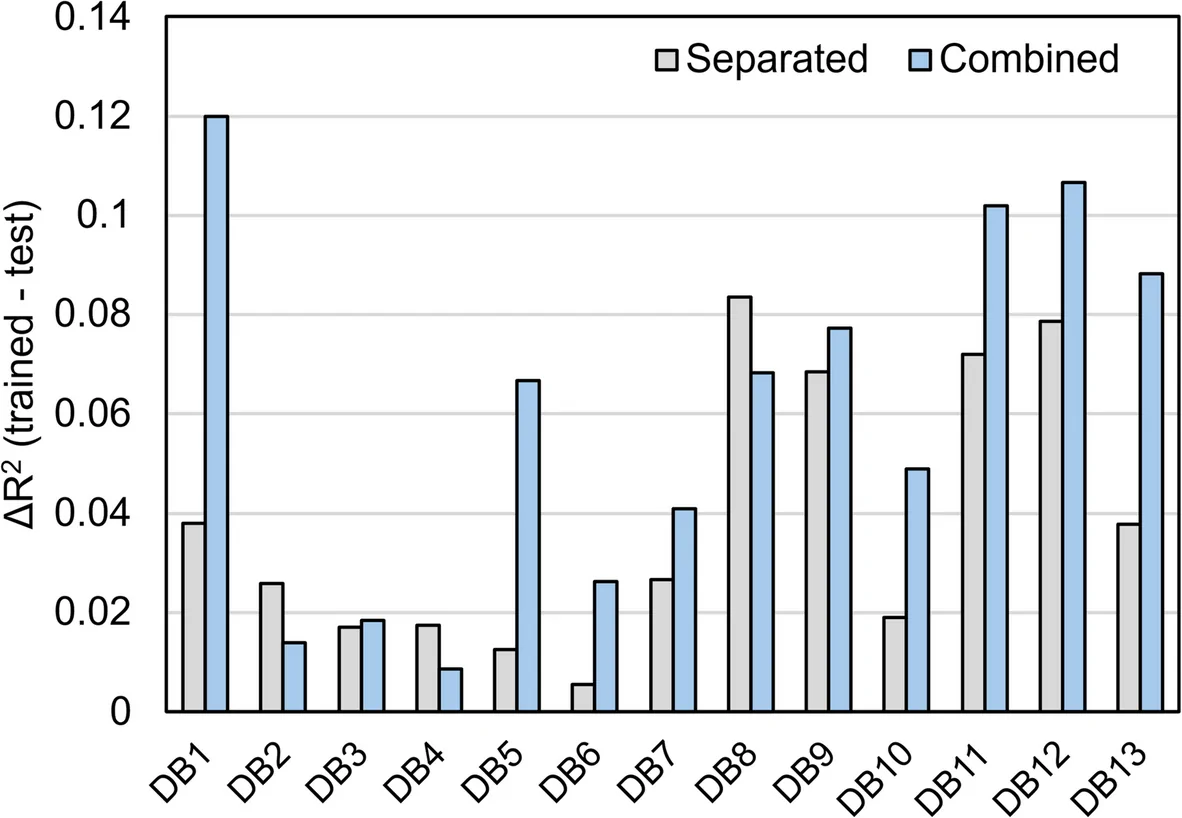
R^2^ difference between trained and test datasets for separated and combined SCM modeling.

Figure 2 presents the performance discrepancy between the training and test datasets, referred to as the generalization gap, for both the individual and combined SCM scenarios. Generally, an increase in the number of input variables raises model complexity, potentially leading to overfitting where the model becomes excessively optimized to the training data. However, the results of this study demonstrate an encouraging trend that contradicts this common assumption.

In most databases, the individual SCM scenario recorded a lower generalization gap compared to the combined scenario. The simultaneous improvement in predictive accuracy (as shown in Fig. 1) and the reduction of the generalization gap indicate that the performance enhancement of the individual SCM models may be strategically valid. These findings indicate that integrating SCMs with diverse chemical compositions into a single variable could act as noise, whereas providing independent information for each material tends to enable the model to better capture and generalize latent patterns in the data. Thus, the individual featurization strategy can be considered as an effective data processing approach, as it appears to satisfy both predictive precision and model stability.

**Fig. 3:**
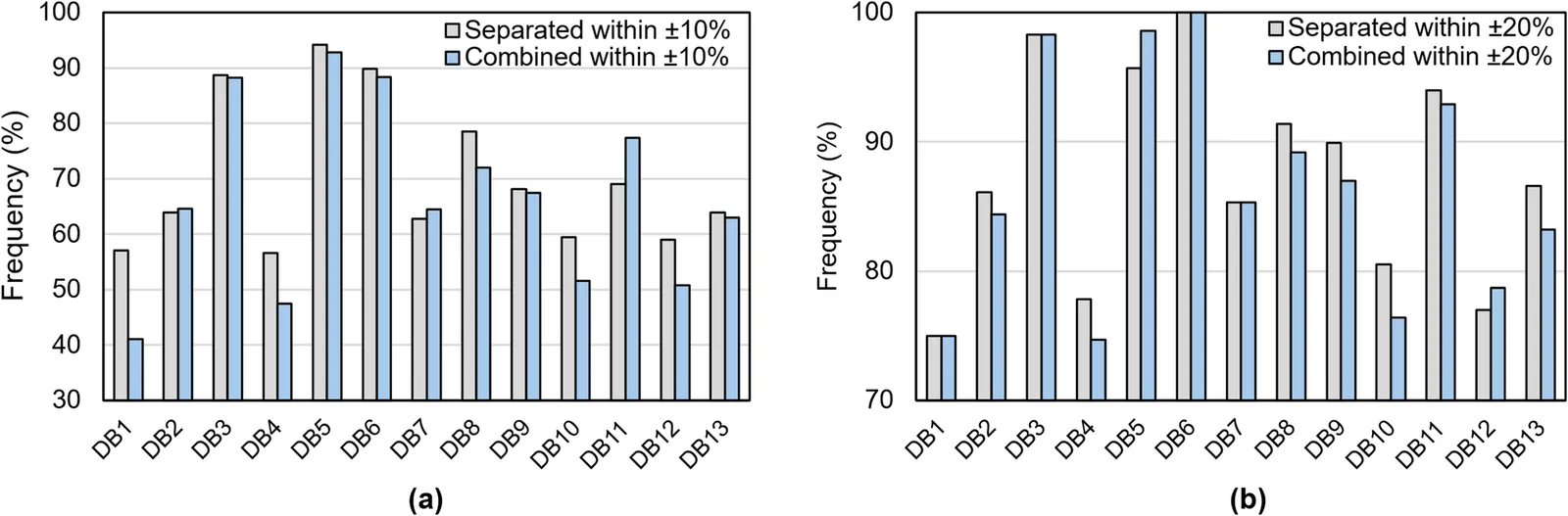
Frequency of predicted samples within specific error bounds: (**a**) samples within ± 10% and (**b**) ± 20% error range.

Figure 3 presents the frequency analysis of prediction errors to further examine the precision and reliability of the model predictions. Figures 3a, b show the frequencies within the ± 10% and ± 20% thresholds, respectively. It should be noted that the y-axis scales were set independently to clearly contrast the sample distributions within each range. Detailed error distribution histograms for each of the 13 databases are provided in Appendix B. Regarding the proportion of samples within the ± 10% error range (Fig. 3a), the individual SCM models recorded higher frequencies than the combined models in most databases. For DB1 (from 41.3 to 57.0%), DB4 (from 47.3 to 56.5%), and DB10 (from 51.6 to 59.4%), the individual featurization allowed more samples to fall within this high-precision range. This superiority was generally maintained in the analysis based on the ± 20% error threshold (Fig. 3b). Specifically, for DB10, the proportion of samples within this range rose from 76.5% in the combined model to 80.6% in the individual model, thereby enhancing prediction stability. Although minor differences or slight reversals were observed in a few cases, such as DB11 and DB12, the overall trend suggests that utilizing individual SCM is more advantageous for improving and stabilizing the model reliability.

To verify that the performance improvement of the individualized SCM featurization strategy is not a unique characteristic limited only to a specific boosting architecture, a cross-algorithmic validation was performed using the RF algorithm. As summarized in Table 5, a similar trend was observed. Although there is no consistent consensus on what extent of increase in R^2^ can be considered an improvement, assuming differences of less than 1% as equivalent, the individualized variable strategy showed a distinct performance improvement in 6 DBs (DB1, DB2, DB4, DB5, DB6, and DB10). In the other 7 databases, the two strategies showed similar and equivalent performance. Most notably, based on the independent test set results, there was not a single database where the combined variable approach achieved an R^2^ higher than the individualized strategy by more than 1%.

**Table 5:** Predictive performance of random forest models under two SCM scenarios.

No	Set	R^2^		RMSE		MAE		MAPE	
		Combined	Individual	Combined	Individual	Combined	Individual	Combined	Individual
DB1	Train	0.770	**0.882**	7.77	5.68	5.37	4.37	26.0	18.9
	Test	0.857	**0.910**	7.43	5.88	5.69	4.56	28.6	20.8
DB2	Train	0.865	**0.876**	5.82	5.58	4.19	4.02	14.7	13.9
	Test	0.879	**0.893**	5.89	5.54	4.21	3.99	15.3	14.3
DB3	Train	0.945	0.941	9.28	9.53	6.72	6.93	6.0	6.2
	Test	0.957	0.955	8.48	8.71	6.06	6.38	6.3	6.6
DB4	Train	0.795	**0.811**	12.04	11.22	8.30	7.71	15.8	14.8
	Test	0.802	**0.832**	12.65	11.64	8.92	8.31	16.2	14.9
DB5	Train	0.846	**0.884**	4.24	3.66	3.27	2.80	6.9	6.0
	Test	0.899	**0.922**	3.84	3.37	3.12	2.62	6.7	5.6
DB6	Train	0.786	**0.838**	0.41	0.36	0.32	0.27	7.0	6.0
	Test	0.799	**0.854**	0.47	0.40	0.35	0.28	7.4	5.8
DB7	Train	0.808	0.815	6.79	6.68	5.09	5.01	13.7	13.5
	Test	0.820	0.828	7.09	6.94	4.88	4.82	13.6	13.3
DB8	Train	0.744	0.738	4.03	4.08	3.07	3.12	10.1	10.3
	Test	0.819	0.821	3.53	3.51	2.44	2.44	8.3	8.3
DB9	Train	0.705	0.714	0.48	0.47	0.36	0.35	11.2	11.1
	Test	0.765	0.775	0.46	0.45	0.36	0.35	12.4	12.1
DB10	Train	0.753	**0.778**	8.74	8.26	6.16	5.66	15.8	14.3
	Test	0.789	**0.822**	8.48	7.79	6.24	5.59	16.8	14.7
DB11	Train	0.776	**0.835**	1.09	0.95	0.74	0.67	15.3	14.6
	Test	0.861	0.864	0.83	0.83	0.57	0.60	9.3	9.9
DB12	Train	0.713	0.678	4.57	4.80	3.21	3.42	11.5	12.3
	Test	0.587	0.588	5.49	5.48	4.26	4.21	12.1	11.9
DB13	Train	0.767	0.777	0.62	0.60	0.42	0.44	12.7	13.1
	Test	0.819	0.804	0.53	0.55	0.40	0.42	13.1	13.3

Significant values are bold.

To prove the robustness of the derived results, the fivefold cross-validation process was repeated five times using independent random seeds, and the results are summarized in Table 6. As shown in the table, the individualized featurization strategy improved predictive accuracy compared to the combined strategy in both the XGBoost and RF models. For the XGBoost models, the individualized strategy achieved a higher mean test R^2^ in 9 out of the 13 DBs, while the remaining 4 DBs showed an R^2^ difference of less than 1%. For the RF models, 8 DBs exhibited higher accuracy under the individualized strategy, and the remaining databases showed differences of less than 1%.

**Table 6:** Predictive performance from fivefold cross-validation repeated five times with different random splits.

No	Variable	XGB		RF	
		Mean R^2^	Std Dev	Mean R^2^	Std Dev
DB1	Combined	0.859	0.060	0.809	0.055
	Individual	**0.906**	**0.037**	**0.860**	**0.049**
DB2	Combined	0.922	0.009	0.885	0.013
	Individual	0.929	0.008	**0.895**	**0.009**
DB3	Combined	0.965	0.004	0.945	0.010
	Individual	0.964	0.005	0.948	0.008
DB4	Combined	0.857	0.036	0.860	0.031
	Individual	**0.874**	**0.029**	**0.870**	**0.031**
DB5	Combined	0.948	0.012	0.869	0.030
	Individual	0.952	0.017	**0.890**	**0.028**
DB6	Combined	0.856	0.019	0.797	0.036
	Individual	**0.907**	**0.015**	**0.862**	**0.038**
DB7	Combined	0.837	0.023	0.809	0.034
	Individual	**0.852**	**0.022**	0.815	0.032
DB8	Combined	0.821	0.041	0.760	0.046
	Individual	0.824	0.045	0.759	0.048
DB9	Combined	0.757	0.038	0.722	0.028
	Individual	**0.783**	**0.035**	**0.736**	**0.027**
DB10	Combined	0.777	0.009	0.752	0.015
	Individual	**0.832**	**0.009**	**0.784**	**0.010**
DB11	Combined	0.825	0.066	0.801	0.045
	Individual	**0.863**	**0.038**	**0.844**	**0.033**
DB12	Combined	0.684	0.055	0.685	0.024
	Individual	**0.726**	**0.018**	0.692	0.012
DB13	Combined	0.799	0.022	0.771	0.022
	Individual	**0.836**	**0.028**	0.779	0.019

Significant values are bold.

A key observation is the change in the standard deviation. When using the individualized strategy, the standard deviation in most databases either remained comparable or decreased. For example, in the XGBoost results for DB1, the combined approach showed a standard deviation of 0.060 depending on the data split, but the individualized strategy reduced this variance to 0.037, demonstrating enhanced prediction stability. These results indicate that the effect of individualizing SCM-related variables is not due to a lucky data split, but rather demonstrates that the models robustly capture the impact of each SCM on the output.

To test the statistical differences between the individualized SCM featurization and the combined approach, the Wilcoxon signed-rank test was performed based on the mean R^2^ scores for both the XGBoost and RF models. The significance level for the Wilcoxon signed-rank test was established at 0.05. According to this threshold, a p-value lower than 0.05 rejects the null hypothesis, which posits that the predictive capabilities of the two approaches are statistically identical. The statistical analysis yielded a p-value of 0.00403 for the XGBoost models and 0.00854 for the RF models, confirming that utilizing SCMs as individualized variables leads to a statistically significant enhancement compared to integrating them into a single combined approach.

### SHAP

This section elucidates the internal decision-making mechanisms of the models using SHAP analysis. Specifically, feature contributions were analyzed for five databases (DB3, DB4, DB5, DB10, and DB12) that exhibited distinct data characteristics or significant differences in predictive performance.

**Fig. 4:**
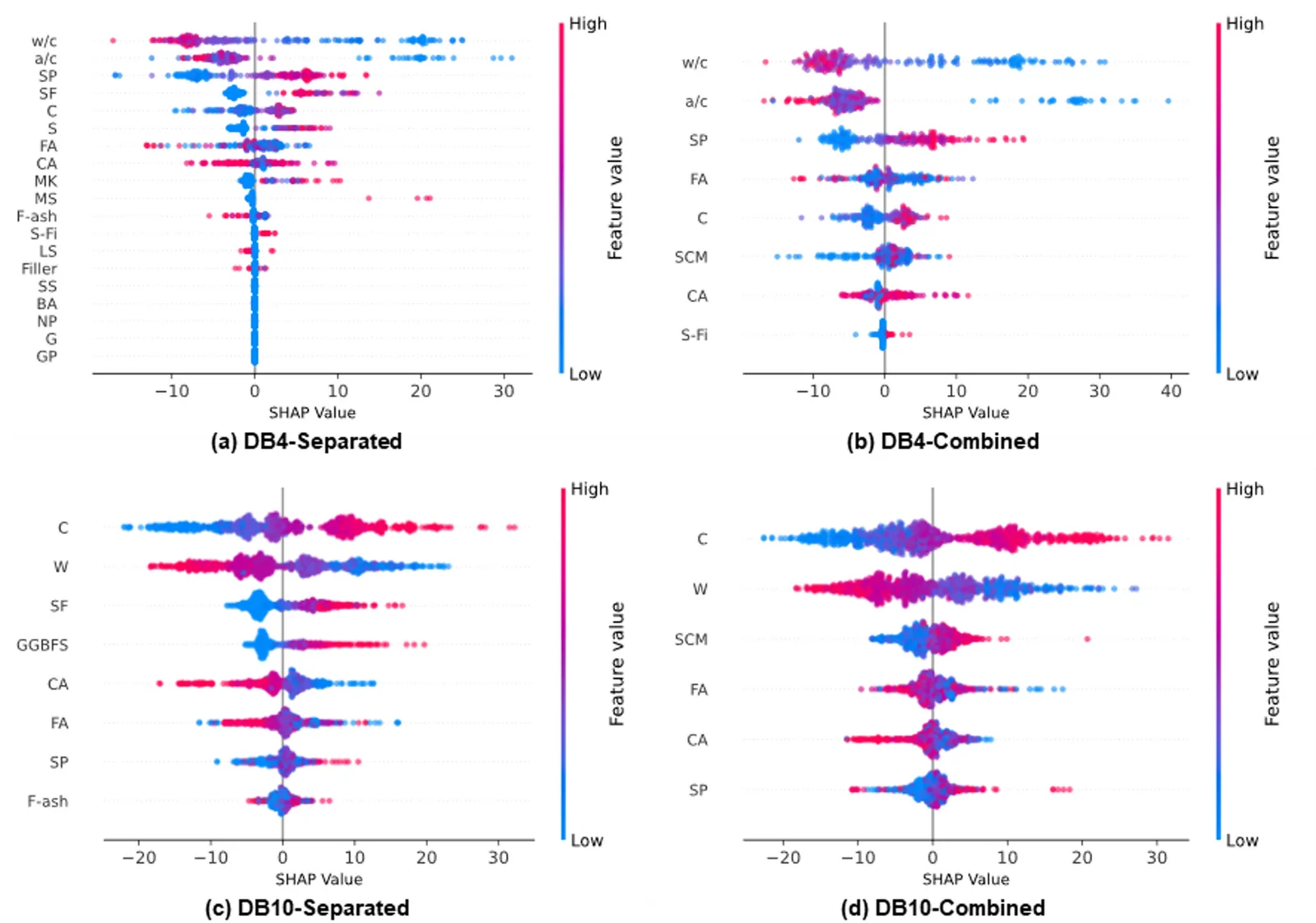
SHAP summary plot: (**a**) DB4-separated; (**b**) DB4-combined; (**c**) DB10-separated; (**d**) DB10-combined.

Figure 4 shows the SHAP summary plots for DB4 and DB10. These cases represent databases where the generalization gap was most substantially reduced when SCMs were treated as individual variables rather than integrated ones. DB4 features a complex dataset containing 11 different types of SCMs. Analysis of the individual variable model (Fig. 4a) confirmed that each SCM component exerts a distinct influence on CS. Specifically, SF, MK and MS showed a clear positive correlation, with higher contents leading to positive SHAP values, meaning that these SCMs contribute to CS enhancement. In contrast, F-ash tended to decrease strength, as its higher content resulted in SHAP values distributed primarily in the negative region. Meanwhile, LS and filler did not show a consistent influence, and components such as SS, BA, and NP had SHAP values concentrated near zero, indicating negligible impact. This is likely because the limited number of samples containing these specific SCMs made it difficult for the model to learn meaningful patterns. These conflicting influences among components become a primary cause of performance degradation in the combined model (Fig. 4b). By grouping the effects of strength-enhancing (SF, MK, etc.) and strength-decreasing (F-ash, etc.) components into a single SCM variable, the model causes these opposing physical effects to cancel each other out. Consequently, the model fails to capture the clear relationship between the total SCM dosage and the actual CS, leading to lower precision and poor generalization. A similar trend is observed in DB10. In the individual model (Fig. 4c), the model clearly distinguishes and learns the independent and positive contributions of SF and GGBFS to CS, while also capturing the inconsistent reactivity of F-ash. However, in the combined model (Fig. 4d), this complexity is lost, and the model merely represents the relationship as a simple positive correlation between the SCM and the SHAP value.

**Fig. 5:**
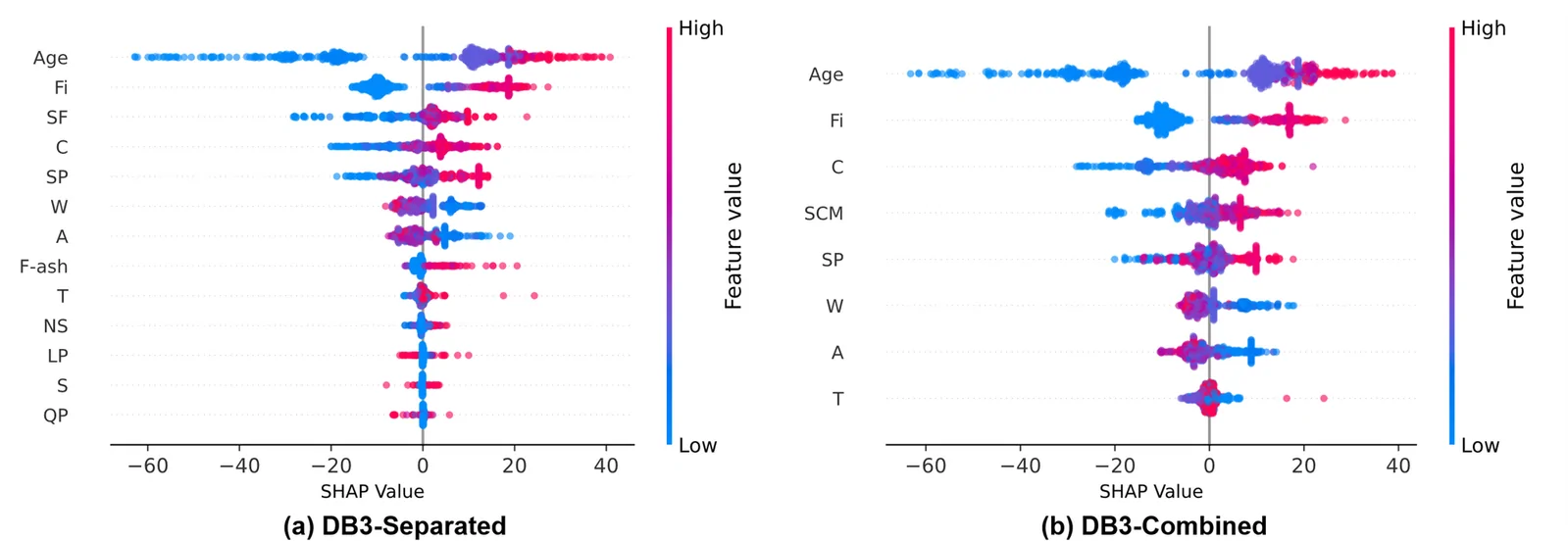
SHAP summary plot: (**a**) DB3-separated; (**b**) DB3-combined.

In the case of DB3, which recorded a R^2^ of over 0.96, the performance difference according to the SCM processing strategy was negligible, unlike the previous analysis results. In both scenarios (Figs. 5a, b), Age and Fi were identified as the most dominant variables for CS prediction. A notable feature of the SHAP analysis for DB3 models is the overwhelming influence of the Age variable, its SHAP value range (− 60 to 40) is significantly broader than those of other databases (approximately − 20 to 20). This suggests that the model assigns an extremely high weight to Age when determining strength development. Under such conditions, even if individual SCMs like LP, S, or QP make contributions, their effects may be overshadowed by the vast variations attributed to Age, preventing them from showing clear directional significance in SHAP values. Consequently, the dominant factor, Age, governs the decision logic of the model, hence, the featurization strategy for SCMs does not appear to induce fundamental changes in the overall decision-making structure.

**Fig. 6:**
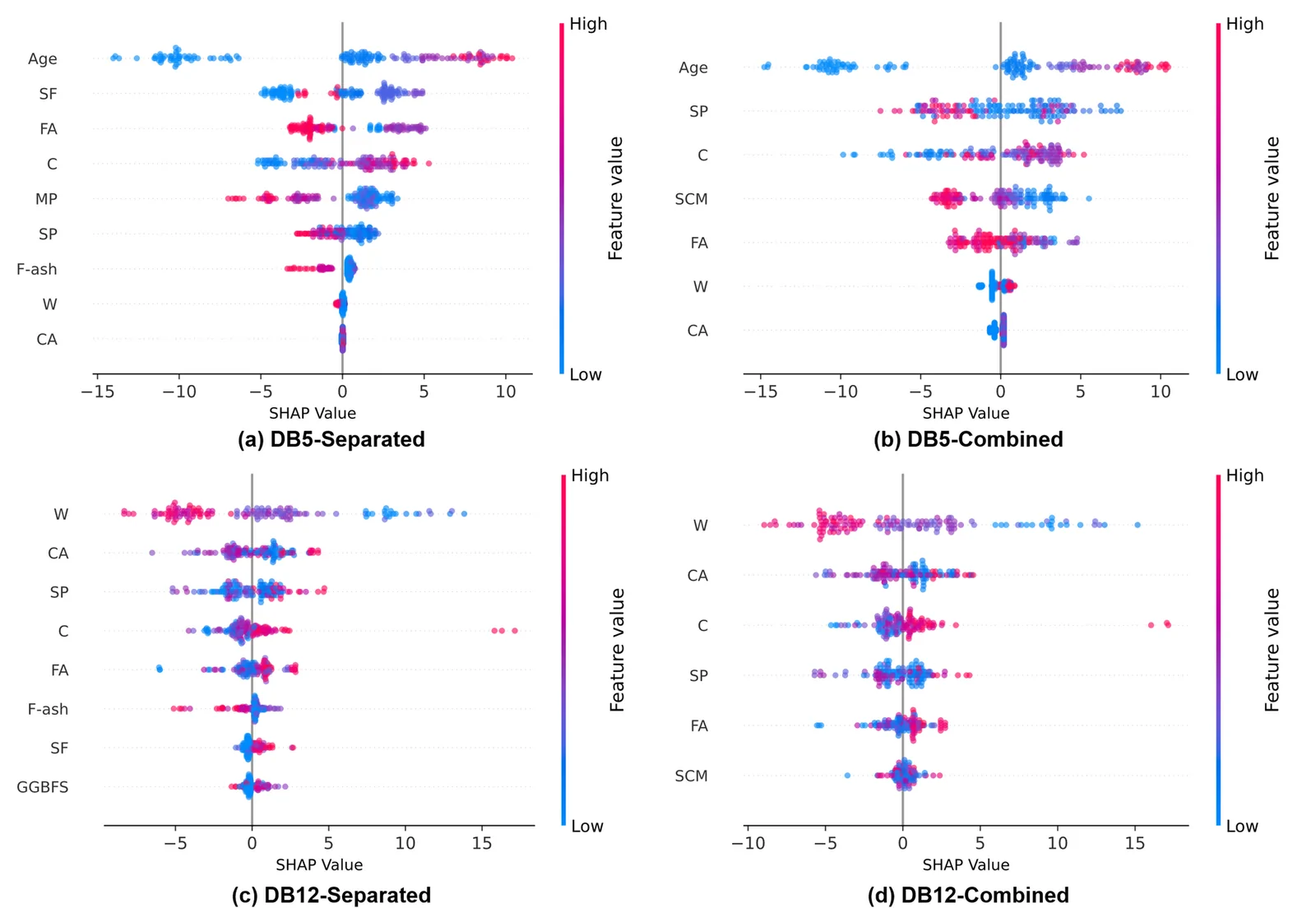
SHAP summary plot: (**a**) DB5-separated; (**b**) DB5-combined; (**c**) DB12-separated; (**d**) DB12-combined.

Figure 6 presents the SHAP summary plots for DB5, which recorded the lowest MAPE, and DB12, which exhibited lowest predictive performance. A comparison of these two databases with contrasting performance provides in-depth insights into how individual SCM featurization affects model interpretability and precision.

DB5 represents a case that achieved exceptionally high predictive accuracy regardless of the SCM processing strategy. Analysis of the individual model (Fig. 6a) reveals that both primary SCM components, MP and F-ash, exhibit the same directional contribution; higher contents are associated with negative SHAP values, indicating a reduction in strength. Since the incorporated SCMs share a clear pattern of consistent negative influence on CS, the combined model (Fig. 6b) could readily learn the relationship that an increase in total SCM leads to a decrease in CS. Consequently, the strong performance in the DB5 models can be attributed to minimal information interference within the data, allowing the model to establish stable decision logic in both featurization approaches.

In contrast, DB12, which recorded the lowest R^2^, clearly illustrates the limitations of integrated variable processing through SHAP analysis. In the individual model (Fig. 6c), there is a complex mixture of F-ash (negative correlation), SF (positive correlation), and GGBFS (inconsistent directionality). In such an environment where material contributions conflict with each other, the combined model (Fig. 6d) results in these differing physical effects canceling out or being diluted. Accordingly, the model could not clearly grasp the effect of SCM on CS.

## Discussion

The findings of this study indicate that inputting SCMs as independent variables generally has a favorable influence on predictive performance. However, it should be noted that this may not necessarily be the optimal strategy for every scenario. In cases where the dataset size is limited or SCM components share a consistent correlation (e.g., DB5), the combined SCM approach can simplify the model structure, potentially leading to more robust performance.

Furthermore, the SHAP results suggest that the influence of a specific SCM observed in a single experiment may not be universally applicable in a multi-source dataset environment. For instance, F-ash acted as a strength-reducing factor in DB4 and DB10 but was observed as a strength-enhancing factor in DB3. These discrepancies arise because the databases typically used for developing ML models in concrete performance prediction are taken from diverse experimental sources; thus, they should be interpreted as the result of complex interactions between different laboratory environments, operator proficiency, and material properties.

## Conclusions

This study investigated the influence of integrating SCMs as either a single combined variable or individual components on the predictive performance ML models. The primary conclusions are as follows:

1. The separated SCM strategy achieved higher R^2^ and lower errors across most databases compared to the combined approach. Furthermore, the individual SCM featurization reduced the performance gap between the training and testing sets, enhancing the generalization ability.
2. Frequency analysis showed that the proportion of samples within the ± 10% and ± 20% error ranges increased for the individual SCM models.
3. SHAP analysis confirmed that when SCM components have opposing influences on strength, integrating them into a single variable causes their effects to cancel out, creating informational noise. Conversely, the separated strategy allows the model to independently recognize each SCMs contribution.

Although this study proposes an optimal featurization strategy for SCMs, it represents only a single step in the ongoing effort to establish robust ML methodologies for complex engineering problems. Despite the improved predictive performance of the individualized SCM strategy in many cases, this specific trend is valid only within the scope of the 13 databases and two ML algorithms evaluated in this study. Therefore, further validation using more diverse and extensive datasets is required to ensure universal applicability. Furthermore, future research should focus not only on algorithmic improvements but also on advanced feature engineering that better reflects the physical significance of the data. Such efforts will facilitate an optimal equilibrium between data complexity and model interpretability, leading to more sophisticated and reliable concrete performance prediction systems.

## Supplementary Information

Below is the link to the electronic supplementary material.

Supplementary Material 1

Supplementary Material 2

## Data Availability

All data generated or analysed during this study are included in this published article and its supplementary information files.
