# Supplementary material for: Featurization strategies of supplementary cementitious materials for enhancing interpretability and precision in machine learning based concrete strength prediction
Source: Sci Rep. 2026 Jun 11;16:26757. doi: 10.1038/s41598-026-57522-1 (PMC13507166; doi:10.1038/s41598-026-57522-1)
Supplement: Supplementary file 1 [file 41598_2026_57522_MOESM2_ESM.docx]

**Featurization strategies of supplementary cementitious materials for enhancing interpretability and precision in machine learning based concrete strength prediction**

**Figure B1. Scatter plots of different SCM featurization (left) and corresponding error histograms (right) for DB1–3**

**Figure B2. Scatter plots of different SCM featurization (left) and corresponding error histograms (right) for DB4–6**

**Figure B3. Scatter plots of different SCM featurization (left) and corresponding error histograms (right) for DB7–9**

**Figure B4. Scatter plots of different SCM featurization (left) and corresponding error histograms (right) for DB10–13**
